# Feasibility of preimplantation genetic testing for aneuploidy on frozen-thawed embryos following conventional IVF insemination

**DOI:** 10.3389/fendo.2024.1441014

**Published:** 2024-10-01

**Authors:** Xiaojun Wen, Zhiming Li, Lizi Cheng, Junye Huo, Wenjuan Yu, Zhanhui Ou, Nengqing Liu, Jieliang Li, Xiaowu Fang, Xiufeng Lin

**Affiliations:** ^1^ Reproductive Medicine Center, Boai Hospital of Zhongshan Affiliated to Southern Medical University, Zhongshan, China; ^2^ The Second School of Clinical Medicine, Southern Medical University, Guangzhou, Guangdong, China

**Keywords:** PGT-A, conventional IVF insemination, ICSI, frozen-thawed embryos, biopsy, parental contamination

## Abstract

**Objective:**

Intracytoplasmic sperm injection (ICSI) is commonly employed in preimplantation genetic testing (PGT) to minimize the risk of foreign sperm DNA contamination. Cryopreserved embryos from patients with recurrent miscarriage or repeated implantation failure, who have undergone conventional *in vitro* fertilization (IVF), can be thawed and biopsied for PGT. Therefore, we aimed to assess the accuracy and effectiveness of preimplantation genetic testing for aneuploidy (PGT-A) on frozen embryos using conventional IVF (c-IVF) insemination methods.

**Methods:**

From January 2021 to November 2023, our center conducted 107 thawed cryopreserved embryo biopsy cycles to screen for PGT-A. Among them, 58 cycles used c-IVF insemination, and 49 used ICSI insemination. Basic patient clinical information, laboratory data, PGT test results, and clinical outcome data were collected. To minimize the confounding effects of patient characteristics and embryo quality on PGT-A outcomes, clinical outcomes, and contamination assessment, these variables were included in the analysis. We then evaluated the blastocyst euploidy rate, clinical outcomes, and accuracy of PGT-A results between the two groups and analyzed potential contamination in the c-IVF insemination group.

**Results:**

A total of 320 blastocysts underwent PGT-A testing, with 179 blastocysts from c-IVF insemination and 141 from ICSI insemination. Considering participants’ baseline characteristics and embryological outcomes, no significant differences were found between the two groups regarding infertility type, average age, body mass index, percentage of PGT-A indications, or quality of embryonic development. Regarding PGT-A results, all 320 biopsy samples were successfully analyzed, showing no statistical variance in chromosomal euploidy, abnormality, or mosaicism rates between the two insemination methods. No parental contamination was detected in the c-IVF insemination group. When assessing clinical outcomes, parameters such as biochemical pregnancy, clinical pregnancy, and miscarriage rates did not exhibit significant discrepancies between the two groups, and no misdiagnoses were reported during the study period.

**Conclusion:**

Embryo transfer and PGT-A results are not affected by potential parental contamination in frozen-thawed embryos conceived via c-IVF. PGT-A guided embryo transfer in thawed embryos conceived by c-IVF is a viable and clinically effective approach.

## 1 Introduction

Preimplantation genetic testing (PGT) has rapidly advanced in terms of technology and application. Initially utilized to prevent the vertical transmission of monogenic diseases, it is now predominantly employed for selecting chromosomally normal embryos with higher implantation potential, thereby reducing the risk of transferring genetically abnormal embryos (1, 2).

ICSI has been widely used in PGT. Including the Preimplantation Genetic Diagnosis International Society (PGDIS), the American Society for Reproductive Medicine (ASRM), and the European Society of Human Reproduction and Embryology (ESHRE) PGT Consortium, specialized societies recommend the use of ICSI for PGT (3–5). It was well known that spermatozoa and cumulus cells could adhere to the zona pellucida (ZP), which was major contamination factors during PGT procedures. Wilton et al. summarized the causes of misdiagnosis in PGD, in which ICSI must be performed for all PCR diagnosis to avoid paternal contamination and cumulus cells must be removed prior to biopsy to avoid maternal contamination (2). ICSI was recommended for all PCR cases to reduce the chance of paternal contamination from extraneous sperm attached to the zona pellucida or non-decondensed sperm within blastomeres (6, 7). Lynch, C et al. investigated the necessity of using ICSI in preimplantation genetic testing (PGT), particularly concerning the risk of paternal cell contamination, concluding that using c-IVF as a fertilization method in PGT-A presents a low risk of paternal cell contamination (8). However, the extensive use of ICSI in ART for non-male factor infertility has been controversial. The current available studies that compare ICSI and c-IVF in couples with males presenting with normal total sperm count and motility, show neither method was superior to the other, in achieving live birth, adverse events (multiple pregnancy, ectopic pregnancy, pre-eclampsia and prematurity), also alongside secondary outcomes, clinical pregnancy, viable intrauterine pregnancy or miscarriage (9). Paffoni et al. found that ICSI was associated with lower live birth rates compared to c-IVF when ART was indicated solely due to female factors. Additionally, the use of ICSI in unexplained infertility cycles did not demonstrate a significant improvement (10). Wang et al. found that ICSI did not improve the live birth rate after the first transplant compared with c-IVF in patients with non-severe male factor infertility, although the mean ovum fertilization rate was significantly higher in the ICSI group than in the c-IVF group (75.0% vs. 66.7%). In addition, the study found no significant differences between the two groups in secondary outcome measures, such as fertilization failure, miscarriage, preterm birth, and low birth weight (11). The ESHRE Add-ons working group recommends that the application of ICSI in clinical practice should be carefully tailored to specific circumstances and patient needs, rather than being used as an additional option in terms of cost and indication (12). The analysis data indicates that the average cost of an ICSI cycle is £226 higher than that of an c-IVF cycle (13). In practical IVF procedures, compared with c-IVF, ICSI involves a more complex operational process, placing higher demands on personnel and equipment.

Currently, PGT utilizes amplification techniques, specifically whole-genome amplification (WGA) of trophectoderm (TE) biopsy cells. Recent research findings indicate that during the WGA of TE biopsy cells in fresh PGT cycles using c-IVF, sperm DNA cannot be amplified. The levels of paternal and maternal contamination are extremely low and nearly negligible, suggesting that the likelihood of misdiagnosis is minimal (14–16). Huang et al. and Dong et al. demonstrated the feasibility of PGT-A under c-IVF by establishing a method for analyzing parental contamination (17, 18). Zhang et al. retrospectively analyzed pregnancy outcomes in frozen PGT cycles of patients who primarily experienced abortion with chromosomal aberration or recurrent implantation failure after c-IVF or ICSI treatment and observed that the rates of clinical pregnancy, early miscarriage, and ongoing pregnancy were similar between the two groups of insemination methods (19). In cases of repeated implantation failures or miscarriages attributed to chromosomal abnormalities in c-IVF cycles, performing PGT-A on frozen embryos is considered necessary. It is noteworthy that frozen embryos not initially intended for PGT may contain sperm and cumulus cells attached to the zona pellucida. Presently, there is a paucity of studies addressing the amplification of sperm or cumulus cells post-freezing in liquid nitrogen and its potential implications for PGT-A outcomes. However, more research evidences are needed on the feasibility of PGT of frozen embryos fertilized with c-IVF to prove safety and effectiveness. The Individual of pregnant woman and quality of transplanted embryo was not controlled in previous studies. This study aims to evaluate the impact of PGT-A testing using c-IVF frozen embryos, examining the influence of c-IVF sperm or cumulus cells on PGT-A results and exploring the potential utility of this approach in PGT-A cycles.

## 2 Materials and methods

### 2.1 Ethics approval

This study was approved by the Ethics Committee of Reproductive Medicine of Zhongshan Boai Hospital. A total of 107 couples signed consent forms that were approved by the local ethics committee. Of these, 58 couples underwent c-IVF insemination and 49 underwent ICSI insemination. The frozen embryos were subsequently thawed and biopsied for PGT-A testing.

### 2.2 Study design and patients

In this study, we reviewed 107 cycles of PGT-A testing of frozen embryo biopsies performed between January 2021 and November 2023. The study included patients with common indications for PGT-A, such as advanced age (≥38 years old), repeated implantation failure (three or more times), and recurrent miscarriage (two or more times). The study was divided into two main groups: one involved frozen embryos inseminated using c-IVF, with peripheral blood samples from both partners extracted for linked single-nucleotide polymorphism (SNP) analysis to detect embryo contamination; the other involved frozen embryos inseminated using ICSI, a recommended PGT-A insemination method owing to minimal contamination from parental sources, where embryos were only thawed and biopsied for testing. The objectives were to assess euploidy, chromosomal abnormality, mosaicism, biomedical pregnancy, clinical pregnancy, and early miscarriage rates between c-IVF and ICSI frozen blastocysts, and the contamination and detection accuracy of paternal and maternal sources in frozen embryos from c-IVF.

### 2.3 Thawing, biopsy and freezing of embryos

The embryo was vitrified and thawed using RapidWarm™ Cleave reagent (Vitrolife Sweden AB). Thawing solution 1, 2, 3, 4was placed in a 37°C incubator for 30 min for equilibration, whereas thawing solutions 2, 3, and 4 were equilibrated at room temperature for the same duration. After resuscitating the embryos, the front section of the cryotop carrier rod was quickly immersed in pre-warmed thawing solution 1 for 30 seconds, thawing solution 2 for 1 min, thawing solution 3 for 2 min, and thawing solution 4 for 5 min, and finally placed in pre-warmed G2 solution. Following blastocyst recovery, the embryos were cultured for 2–4 h before biopsy. Preheated G-PGD or G-MOPS Plus (Vitrolife, Goteborg, Sweden) was used as biopsy fluid. Under an inverted microscope, the inner cell mass of the blastocyst was identified and fixed at 9–12 o’clock, and the hatched TE cells were aspirated into the biopsy pipette. Three laser pulses of 2.0 ms (ZYLOS-tk^®^, Hamilton Thorne, MA, USA) were used to loosen cell junctions before a mechanical ‘flick’ method was applied to remove 4–10 TE cells. The cells were then washed and placed in a 0.2 mL polymerase chain reaction tube pre-added with 1–2.5 μL phosphate buffered saline for PGT detection. In cases where the blastocyst had not hatched, the laser perforated the zona pellucida for 2.0 ms to induce collapse before the biopsy. After biopsy, the blastocysts were vitrified and frozen within 1 h at room temperature using Vitrification Refrigerant Set (Kitazato Corporation). The vitrification cryogenic solution and the equilibrium solution were equilibrated for 30 min at room temperature. The embryos were aspirated and immersed in an equilibrium solution for 10 min and then the embryos were moved between different drops of vitrification cryogenic solution and rinsed 2-3 times within 60s. After that, the embryos were transferred to a freezing carrier rod and promptly placed in liquid nitrogen for storage.

### 2.4 Whole-genome amplification

WGA was performed as previously described (20) using a ChromSwift kit (Yikon, China) with multiple annealing and looping-based amplification cycles (MALBAC) in accordance with the manufacturer’s protocol.

### 2.5 Determination of blastocyst ploidy status using next generation sequencing

To analyze the ploidy status of the blastocysts, the DNA amplified across WGA-MALBAC was sequenced using a MiSeq sequencer with a single-ended read length of 55 bp. Approximately two million raw reads were generated for each TE biopsy sample. Raw data were analyzed as described previously (20). The original reads were aligned and compared with the UCSC hg19 human reference genome to filter out low-quality and duplicate sequences for copy number variation (CNV) analysis. The R language program was used to visualize the CNVs of the 24 chromosomes. Embryos were diagnosed as euploid when the extent of mosaicism was < 30%. When the CNV was > 4 Mb and the extent of mosaicism was > 70%, the embryo was diagnosed as aneuploid. When the CNV size was > 4 Mb and the extent of mosaicism was between 30% and 70%, the embryo was diagnosed with chromosomal mosaicism.

### 2.6 Determination of parental contamination for c-IVF inseminated embryos

Parental contamination was identified using the quantitative parental contamination test (qPCT), as described by Dong et al. (18). Briefly, the gene SNP method utilizes the Infinium Asian Screening Array bead chip technology (Illumina, Inc., San Diego, CA, USA) to analyze the genotypes of both blastocysts and parents. Following the extraction of peripheral blood DNA from the parents, the WGA DNA of each blastocyst was linearly amplified, fragmented, precipitated, and hybridized according to the manufacturer’s instructions. Subsequently, an iScan system (Illumina, Inc., San Diego, CA, USA) was used to scan the chip signal, enabling analysis of the B allele frequency (BAF) for each sample. The expected BAFs for genotypes AA, AB, and BB were approximately 0, 0.5, and 1, respectively. Hence, if the father’s SNP was BB or AA and the mother’s SNP was AA or BB, the embryo genotype was anticipated to be AB with a BAF of 0.5. Deviations in BAF could indicate parental contamination or biases in alleles that often stem from allelic amplification bias induced by WGA technology. During the data analysis, biased data should initially be disregarded. Ultimately, a BAF exceeding 0.6 or falling below 0.4 suggests parental bias and potential contamination.

### 2.7 Embryo transfer and follow-up

Endometrial preparation was performed using hormone replacement therapy. Blastocysts with normal chromosome copy numbers were thawed and transferred into the uterus five days after progesterone-initiated ovulation. Clinical pregnancy was confirmed if an intrauterine gestational sac was detected by ultrasound along with a fetal heartbeat 30–40 days after frozen embryo transfer (FET). To validate the accuracy of PGT-A results, noninvasive prenatal testing (NIPT) can be performed at 12–18 weeks of gestation or amniocentesis at 16–24 weeks of gestation.

### 2.8 Statistical analyses

Continuous variables are presented as the mean ± standard deviation. Categorical variables are presented as the number of observations and percentages. Differences in variables between the different insemination groups were analyzed using a 2-tailed Student’s *t*-test (female age, body mass index [BMI], infertility type, and blastocyst development on day 5/6/7). The chi-square test was used to analyze the biomedical pregnancy, clinical pregnancy, miscarriage, euploid, abnormal, and mosaic rates and other data (percentage of infertility type, indication for PGT-A, days of blastocyst biopsy, and blastocyst development on day 5/6/7). Statistical significance was set at P <0.05.

## 3 Results

### 3.1 Baseline characteristics and embryological outcomes of the study participants

PGT cycles with at least one successfully resuscitated embryo that could be used for embryo biopsy between January 2021 and November 2022 at our center were reviewed for eligibility. Finally, 107 PGT cycles with embryo biopsies performed after thawing were included in this study. Of these, 58 belonged to the c-IVF insemination group and 49 to the ICSI insemination group. The clinical data of both groups and the embryological data are summarized in **Table 1**.

**Table 1:** Clinical information of the FET cycles and the laboratory data of embryological for each study group.

Characteristics	Study groups		P value (c-IVF vs ICSI)
	c-IVF group	ICSI group	
No. of cycles, n	58	49	–
Infertility type			
Primary infertility, n (%)	9 (15.52%)	10 (20.41%)	0.51
Secondary infertility, n (%)	49 (84.48%)	39 (79.59%)	
Female			
age (years, average ± SD)	35.74 ± 4.33	35.92 ± 5.03	0.25
BMI (kg/m2, average ± SD)	22.31 ± 3.76	22.51 ± 3.17	0.85
The indication for PGT-A			
recurrent abortion, n (%)	23 (39.66%)	19 (38.78%)	
Advanced maternal age, n (%)	24 (41.38%)	20 (40.82%)	0.98
repeated implantation failure, n (%)	11 (18.97%)	10 (20.41%)	
Blastocyst biopsy			
No. of blastocyst, n	179	141	
Blastocyst biopsy on Day5, n (%)	55 (30.73%)	43 (30.50%)	
Blastocyst biopsy on Day6, n (%)	121 (67.60%)	92 (65.25%)	0.41
Blastocyst biopsy on Day7, n (%)	3 (1.68%)	6 (4.26%)	
Blastocyst development on Day5/6/7			
EQ1 (average ± SD)	1.94 ± 1.53	1.87 ± 1.52	0.66
EQ2 (average ± SD)	1.09 ± 1.34	0.92 ± 1.10	0.44
EQ3 (average ± SD)	0.07 ± 0.26	0.08 ± 0.28	0.62
Total EQ1, n (%)	116 (64.80%)	92 (65.25%)	
Total EQ2, n (%)	59 (32.96%)	45 (31.91%)	0.95
Total EQ3, n (%)	4 (2.23%)	4 (2.84%)	

Blastocyst development on Day5/6/7, EQ1 means the embryo score is AA AB BA, EQ2 means the embryo score is BB AC, EQ3 means the embryo score is CA BC CB.

### 3.2 The results of PGT

**Figure 1:**
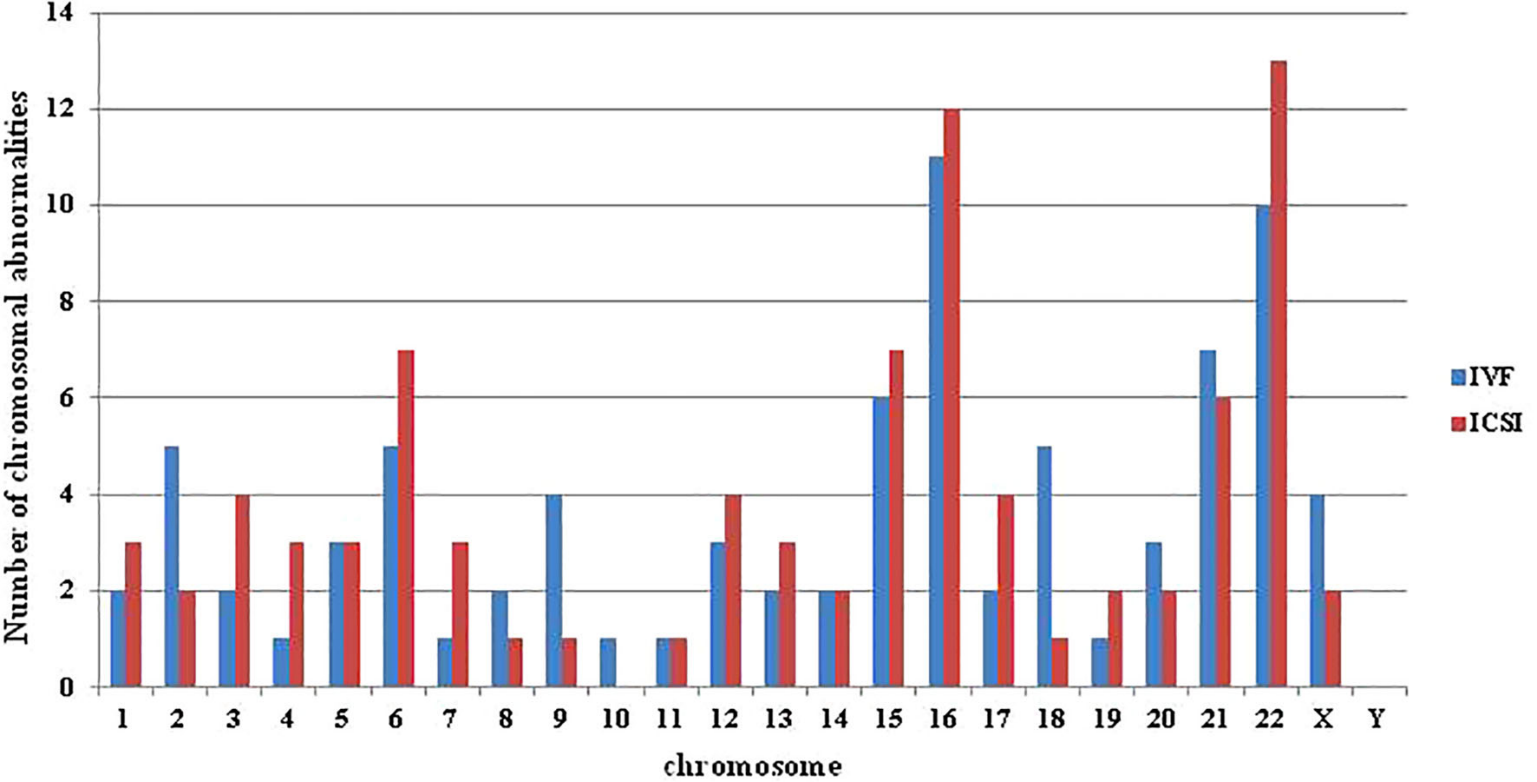
The distribution of abnormalities in each chromosome in the c-IVF and ICSI groups. Abnormalities included chromosome monomer, multibody, and fragment abnormalities, but did not include mosaicism.

A total of 320 blastocysts underwent chromosomal analysis with a 100% success rate for PGT. The molecular karyotype findings were categorized into chromosomal euploidy, chromosomal abnormalities, and chromosomal mosaicism (**Table 2**). There were no statistically significant differences in the rates of chromosomal euploidy (48.60% vs. 50.35%), abnormalities (35.20% vs. 36.88%), or mosaicism (16.20% vs. 12.77%) between the two groups (P = 0.69). **Figure 1** illustrates the distribution of chromosomal abnormalities. Karyotyping results for chromosomal abnormalities were similar between the c-IVF and ICSI insemination groups. Consistent with the findings of previous studies, the most common chromosomal abnormalities were observed on chromosomes 15, 16, 22, and 21. Furthermore, in the c-IVF insemination group, no samples showed parental contamination after combined analysis with parental alleles (contamination rate, 0.00%). Therefore, contamination with paternal DNA-containing sperm and cumulus cells was negligible in thawed embryos inseminated via c-IVF.

**Table 2:** The results of PGT-A in the two study groups.

Item	Study groups		P value (c-IVF vs ICSI)
	c-IVF group	ICSI group	
PCR failure, n (%)	0 (0.00%)	0 (0.00%)	–
Tested embryos			
Euploid, n (%)	87 (48.60%)	71 (50.35%)	0.69
Abnormal, n(%)	63 (35.20%)	52 (36.88%)	
Mosaic, n(%)	29 (16.20%)	18 (12.77%)	
parental contamination, n(%)	0 (0.00%)	0 (0.00%)	–

PCR failure—no embryonal DNA is available for PCR; euploid indicates that the embryo is chromosomally normal and is available for transfer; abnormal indicates that the embryo has CNVs > 4M and mosaicism > 70%; mosaic indicates that the embryo has CNVs > 4M and mosaicism is between 30% and 70%.

### 3.3 Clinical outcomes

According to the PGT results, embryos with the highest morphological score and without chromosomal abnormalities were prioritized for transfer into the uterus. A total of 158 euploid embryos were available for transplantation, with only one embryo transferred in all transfer cycles. The numbers of FET cycles in the c-IVF and ICSI groups were 45 and 39, respectively. **Table 3** presents the primary clinical outcomes of the 84 FET cycles in these groups. Some of the cycles were ongoing pregnancies; therefore, we did not compare the live birth rates. The biochemical pregnancy rate (68.89% vs. 64.10%, P = 0.64), clinical pregnancy rate (57.78% vs. 64.10%, P = 0.55), and miscarriage rate (11.54% vs. 16.00%, P = 0.70) were not significantly different between the c-IVF and ICSI groups. In the c-IVF group, three cases of miscarriage occurred, with one patient undergoing abortion product chromosome testing showing euploidy, whereas the remaining two patients did not undergo testing. The ICSI group had four cases of miscarriage, none of which underwent chromosomal testing for abortion products, and one case was an ectopic pregnancy. No misdiagnoses were observed during the study period.

**Table 3:** Clinical outcomes of the two groups.

Item	Study groups		P value (c-IVF vs ICSI)
	c-IVF group	ICSI group	
FET cycles,n	45	39	–
Biomedical pregnancy rate, n (%)	31 (68.89%)	25 (64.10%)	0.64
Clinical pregnancy rate, n (%)	26 (57.78%)	25 (64.10%)	0.55
Miscarriage rate, n (%)	3 (11.54%)	4 (16.00%)	0.70
The accuracy of PGT-A results, n (%)	23 (100.00%)	21 (100.00%)	–

Biochemical pregnancy is confirmed by elevated levels of human chorionic gonadotropin but does not result in the formation of a pregnancy sac.

Clinical pregnancy refers to B-ultrasound that can find the pregnancy sac.

Miscarriage includes spontaneous abortion and induced abortion.

The accuracy of the PGT-A results indicates the consistency of PGT-A and NIPT or amniocentesis results.

## 4 Discussion

Currently, research on PGT in embryos subjected to c-IVF insemination is limited and its clinical application remains controversial. One primary concern is the potential contamination of biopsy samples with paternal and maternal DNA. Consequently, in routine PGT treatment cycles, the main guidelines and expert consensus of ESHRE, ASRM and PGTIS advocate the use of ICSI as the preferred method of insemination to minimize the risk of contamination from spermatozoa and cumulus cells attached to the embryonic zona pellucida. Considering the off-indication use, potential impact on embryo development and advantages compared with c-IVF, the necessity of ICSI for PGT needs further exploration.

PGT relies on single-cell WGA for detection. The main purpose of WGA technology is to amplify DNA from a small number of biopsy cells, typically 4–10, to generate sufficient DNA templates for subsequent chromosome or gene analysis. Historically, paternal contamination has been a concern in PGT. Various widely used WGA kits are available on the market, including SurePlex, multiple displacement amplification (MDA), MALBAC, and RepSeq. Because of the unique packaging of DNA in sperm, which involves complex structural components, such as the nuclear matrix and small nuclear circles, the chromatin in sperm nuclei is highly condensed. Prior to WGA, lysis with specific reagents, such as proteases or dithiothreitol, is necessary to decondense the sperm chromosomes (21, 22). Some studies suggest that sperm cell amplification may require modified SurePlex or MDA methods before PGT technology can be used for genetic testing of individual sperm. Essentially, when sperm undergo traditional WGA methods, their DNA content may be insufficient for PGT (8). This opens the possibility of using c-IVF insemination methods during PGT cycles.

In a retrospective analysis, Feldman et al. compared the accuracy of c-IVF and ICSI insemination in PGT (14). This study observed comparable PGT results and contamination rates between the two groups, with negligible paternal sperm DNA contamination in the c-IVF insemination group. Additionally, De Munck et al. assessed the effectiveness of c-IVF and ICSI methods in PGT-A cycles involving 30 couples with non-male factor infertility in a prospective study (16). No significant differences were observed between the two groups in terms of fertilization rate, embryo development ability, blastocyst euploidy rate, contamination rate, aneuploid parent source, or uniparental diploid parent source in the PGT cycle. In Zhang et al. study, only one of 150 trophectoderm biopsy samples testing showed maternal contamination level of 10%, and the PGT-A results was validated with 100% consistent by inner cell mass and trophectoderm (19). This further supports the conclusion that c-IVF insemination may be not a risk factor for PGT-A cycle, suggesting that ICSI should be preferred for men with infertility.

The aforementioned studies were preliminary explorations of the use of c-IVF insemination methods in fresh PGT-A cycles. Patients who have previously undergone c-IVF insemination during embryo transfer cycles, often because of factors such as advanced maternal age, recurrent miscarriage, or repeated implantation failure, are now being considered for PGT-A. This test necessitates the thawing and biopsy of frozen embryos. There are two main issues when considering thaw biopsy of frozen embryos from c-IVF insemination. First is that some frozen blastocysts from c-IVF insemination may contain a zona pellucida containing both sperm and cumulus cells. Second is whether sperm and cumulus cells can be easily amplified after freezing in liquid nitrogen. To prevent contamination of biopsy samples with maternal cumulus cells, embryologists meticulously remove cumulus cells from the zona pellucida of the embryo. Additionally, our study utilized the qPCT method described previously (23–25) to assess the susceptibility of frozen embryos inseminated via c-IVF to parental contamination. Recently, researchers conducted a prospective analysis of the risk of parental contamination during PGT-A cycles involving cryopreserved embryos inseminated via c-IVF (18). The contamination rate of 0.83% (1/120) is very close to the 0.00% (0/179) in this study, and no sperm contamination is found. The findings indicated that frozen embryos fertilized with c-IVF undergo PGT-A with minimal risk of contamination from the parents and that MALBAC technology can be effectively utilized for testing frozen embryos resulting from c-IVF in PGT-A cycles.

In the study, the individual and embryonic development characteristics of the patients enrolled in the c-IVF and ICSI groups were analyzed, including female age, BMI, indication, biopsy embryo age and embryo morphology. There was no significant difference between the two groups. Subsequent comparative analysis of PGT-A results and transplantation clinical outcomes between the two groups showed no difference. The research results of Sahin showed that the chromosomal abnormalities rates in embryos fertilized by c-IVF and ICSI were 65% and 69.9% (26), respectively, which were higher than the results of this study. In the Palmerola et al. study, there was no significant difference in the test failure rate (4.4% vs 6.3%), euploidy rate (27.9% vs 30%), aneuploidy rate (45.4% vs 43.1%) and the test failure rate (4.4% vs 6.3%) between the c-IVF and the ICSI group. Though not significant, they identified a trend toward higher rate of mosaicism in IVF (25.9%) versus ICSI (20.9%) (27), which is consistent with the results of this study. De Munck study results showed no significant difference on euploidy rate (49.8% vs 44.1%) between the c-IVF and ICSI PGT-A (16). The findings of Zhang et al. suggested that the choice between c-IVF and ICSI with PGT-A may not significantly impact clinical pregnancy outcomes, as both groups demonstrated comparable pregnancy rates of 59.4% and 55.6%, respectively (19). Interestingly, when comparing the distribution of different chromosomal abnormalities between c-IVF and ICSI with PGT-A in our study, we found a higher prevalence of abnormalities in chromosomes 2, 9, and 18 in the c-IVF group, although the sample size was limited. The discrepancies in PGT-A results and clinical outcomes across different studies can be attributed to various factors, including patient selection criteria, testing methods, and study design.

Although our retrospective study offers support for the use of c-IVF in PGT-A cycles, it has some limitations. This study was limited to 58 c-IVF insemination cycles and a limited number of patients. It is crucial to recognize that PGT-M and PGT-SR involve distinct treatment options and present varying risk profiles. Therefore, our findings should not be extrapolated to patients undergoing PGT-M (monogenic disorders) or PGT-SR (structural chromosomal rearrangements). Moreover, because of the standard treatment process for PGT-A, embryos in the ICSI group were not tested for parental contamination using qPCT, leading to an inability to assess parental contamination in this group. Finally, a key drawback of this clinical application strategy is the requirement for invasive embryo biopsy. The repeated thawing and freezing of embryos may raise safety concerns.

In summary, c-IVF as an insemination method for PGT-A is associated with a lower risk of parental contamination and misdiagnosis. This study demonstrates the feasibility of performing PGT-A on frozen embryos resulting from c-IVF insemination. Clinicians recommend that patients with cryopreserved embryos obtained from c-IVF should consider this option.

## 5 Conclusion

This study verified the efficacy of PGT-A detection in frozen embryos resulting from c-IVF insemination by analyzing the outcomes of PGT on frozen embryos from both c-IVF insemination and ICSI insemination. The findings of this research could potentially be extrapolated to the implementation of PGT-SR or PGT-M in future studies.

## Data Availability

The data that support the findings of this study have been deposited into CNGB Sequence Archive (CNSA) of China National GeneBank DataBase (CNGBdb) with accession number CNP0006266.
